# Quality of life in children with OCD with and without comorbidity

**DOI:** 10.1186/s12955-014-0152-x

**Published:** 2014-10-29

**Authors:** Bernhard Weidle, Thomas Jozefiak, Tord Ivarsson, Per Hove Thomsen

**Affiliations:** Department of Child and Adolescent Psychiatry, St. Olavs University Hospital, Trondheim, Norway; Norwegian University of Science and Technology, Faculty of Medicine, Regional Centre for Child and Youth Mental Health and Child Welfare, Trondheim, Norway; Centre for Child and Adolescent Mental Health, Eastern and Southern Norway, Gullhaug Torg 4B, 0484 Oslo, Norway; Psychiatric Hospital for Children and Adolescents, University Hospital in Aarhus, Risskov, Denmark

**Keywords:** Pediatric OCD, Quality of life, Comorbidity, Assessment

## Abstract

**Background:**

Quality of life (QoL) is a well-established outcome measure. However, in contrast to adult obsessive-compulsive disorder (OCD), little is known about QoL in children with OCD. This study aimed to assess QoL, social competence and school functioning of paediatric patients with OCD by comparing them with the general population and assessing the relations between comorbidity, duration and severity of symptoms, family accommodation and QoL.

**Methods:**

Children and adolescents (n = 135), aged 7–17 (mean 13 [*SD* 2.7] years; 48.1% female) were assessed at baseline for treatment. QoL was assessed by self-report and caregiver’s proxy report on the Questionnaire for Measuring Health-related Quality of Life in Children and Adolescents (KINDL-R) and compared with an age- and sex-matched sample from the general population. Social competence and school functioning were assessed with the Child Behavior Checklist, comorbidity with the Kiddie Schedule for Affective Disorders and Schizophrenia (Present and Lifetime Version), severity of OCD with the Children’s Yale-Brown Obsessive Compulsive Scale and the families’ involvement with the child’s OCD symptoms with the Family Accommodation Scale.

**Results:**

QoL and social competence were reduced (*p* < .001) in patients with OCD compared with controls (KINDL-R mean score 62.40 [*SD* 13.00] versus 69.72 [12.38] in self-reports and 61.63 [*SD* 13.27] versus 74.68 [9.97] in parent reports). Patients with comorbidity had lower QoL (*p* = .001) in proxy ratings than those with OCD only (mean score 56.26 [*SD* 12.47] versus 64.30 [*SD* 12.75]). In parent proxy reports, severity of OCD (*r* = −.28) and family accommodation (*r* = −.40) correlated moderately negatively with QoL.

**Conclusions:**

To our knowledge, this is the largest QoL study of paediatric OCD. QoL was markedly reduced in children with OCD, especially in those with comorbid psychiatric disorders. Based on our findings, we suggest employing QoL assessment in order to have a more comprehensive understanding of childhood OCD.

**Clinical trials registration information:**

This study was registered in Current Controlled Trials; Nordic Long-term Obsessive Compulsive disorder (OCD) Treatment Study (ISRCTN66385119).

## Background

Obsessive-compulsive disorder (OCD) is reported with a prevalence of 0.25% in paediatric cases, with a more frequent distribution (0.6%) in the range between 13 and 15 years of age [1]. Earlier studies suggested higher rates of between 1% and 3% [2,3]. The disorder has a chronic course in 40–75% of cases [4-6]. Between 30% and 50% of adults with OCD report that symptoms started in childhood or adolescence [7]. In the majority of cases, OCD is associated with other psychiatric conditions; as many as 77–85% of children with OCD fulfilled criteria for one or more other psychiatric diagnoses [8,9]. Depression, phobias and other anxiety disorders and neuropsychiatric conditions, such as attention deficit hyperactivity disorder (ADHD) and tic disorder are frequent comorbidities in paediatric OCD. In addition, several researchers have reported elevated levels of autism spectrum disorder comorbidity in OCD, in adult [10-12] as well as in paediatric samples [13].

Quality of life (QoL) assessment is established as an important outcome factor in clinical trials [14], independent from symptom-level assessment, as pathology does not have a simple linear relationship to well-being [15]. Despite a variety of assessment instruments and focuses, most studies of QoL in adult populations agree that QoL is reduced in patients with OCD and that QoL changes seem to be associated with symptom severity [16,17]. A recent review showed that QoL in adults with OCD is significantly impaired, when compared with QoL in the general population [18]. Comorbid conditions, particularly depression, were major contributing factors to the reduced QoL in OCD. The authors underlined the importance of QoL assessment in both clinical and research settings to examine disease burden, to monitor treatment effectiveness, to determine grade of recovery from OCD and to take these factors into account for the development of treatment plans.

In paediatric OCD, there is still a paucity of studies. In the above-mentioned review [18], a systematic literature search on OCD and QoL identified only seven articles concerning children or adolescents, which were subsequently excluded from the review because of the selection criterion of “studies evaluating adults”. Our own search strategy did not reveal additional research papers. A longitudinal cohort study [19] evaluated baseline characteristics of 36 children, obtained at a mean age of 12 years, with regard to their predictive value for QoL in young adulthood after an average of nine years follow-up. QoL measurement was applied at follow-up only. OCD appeared to most strongly impair the interpersonal relationships and work domains of QoL. QoL and severity of OCD and anxiety symptoms were significantly associated in early adulthood. In a long-term follow-up of 142 children and adolescents with OCD over five years [4], the persistence rate of OCD was 41%; functional impairment and QoL were mildly to moderately affected. QoL was assessed at follow-up at a point in time when the mean age of the participants was 18.6 years (*SD* =3.5, range 11–28) and 61% reported very much or much improvement of OCD symptoms. Baseline data were not available. In a survey exploring QoL among members of the Danish OCD Association, about half of the 219 individuals with OCD who completed the self-report questionnaire were beyond age 18 [20]. Because of the nature of the survey, there were several limitations, such as sample selection and lack of formal assessment. Nevertheless, it is noteworthy that 72% of the respondents reported affected social and daily life functioning and 26.5% reported dissatisfaction with their quality of life.

Lack and colleagues [21] assessed baseline QoL in 62 children and adolescents (8–17 years) presenting for an initial treatment evaluation. QoL scores were significantly lower than for healthy controls, and moderately associated with OCD symptom severity as reported by parents. In addition, the presence of comorbid externalizing and internalizing symptoms was a strong predictor for lower QoL scores. The authors described the lack of structured interviews to make the diagnoses of OCD and comorbidities as a limitation. To our knowledge, the present study is the largest QoL study of paediatric patients with OCD and the first one based on the assessment of OCD and comorbid disorders by standardized semi-structured diagnostic interviews.

### Aims

The aims of the present study were: (1) to assess QoL, as reported both by paediatric patients with OCD and their caregivers, compared with an age- and sex-matched sample of student and parent reports from the general population; (2) to compare the social competence and school functioning of these patients with that of the general population; (3) to investigate the relations between comorbid disorders such as ADHD, Tourette’s syndrome, other anxiety disorders, depression and QoL; and (4) to explore the significance of other factors for the perceived quality of life, such as duration, severity, and the family’s adjustment to the OCD symptoms.

## Methods

### Participants

**Figure 1:**
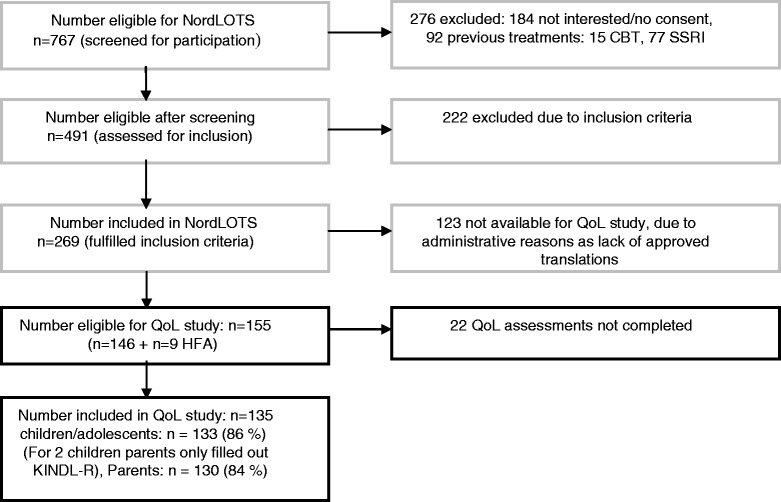
**Flow chart.** NordLOTS main study (boxes with a grey border) and QoL study (boxes with a black border). Abbreviations: NordLOTS = Nordic Long-term Obsessive-compulsive disorder Treatment Study; QoL = Quality of Life; HFA = High functioning autism; KINDL-R = Questionnaire for Measuring Health-related Quality of Life in Children and Adolescents; CBT = Cognitive behaviour therapy; SSRI = Selective Serotonin Reuptake Inhibitor.

In the framework of a multicentre treatment research project (the Nordic Long-term Treatment Study [22]), QoL was assessed in children and adolescents 7–17 years of age, diagnosed with OCD and presenting for a baseline evaluation between September 2008 and June 2012. The present QoL study was initiated later than the Nordic Long-term Treatment Study and at different time points in the three participating countries, due to availability and approval of the national translations of the QoL questionnaire used. The Danish translation was prepared during the inclusion process and approved by the original authors only in March 2011. The first patients available for QoL assessment were included in September 2009 in Norway, in August 2010 in Sweden and in April 2011 in Denmark, leading to a total of 135 participants with the following distribution: 76 participants (56.3%) from Norway, 33 (24.4%) from Sweden and 26 (19.3%) from Denmark. Details of patient characteristics and results of the Nordic Long-term Treatment Study are described elsewhere [23] (Figure 1).

### Comparison group

A large norm data sample consisting of students from schools in Sør-Trøndelag county [24], representing a comparable geographical area with both urban and rural settlement, was used as a control group. Every child or adolescent with OCD was individually matched to a student from the general population sample (n =1821, 8–16 years). This sample was stratified according to sex and age, and students were consecutively numbered in each stratum. Using computer-generated random numbers, we then allocated each patient to a control. With regard to parent education, there was no significant difference between patients (*M* = 5.19) and the allocated students from the general population (*M* = 5.29) (*t*(118) = .51, *p* = .61, paired samples *t* test).

Because of the cluster design of the general population study (sampling data from grades 4, 6, 8 and 10), we had to stratify the samples before matching patients and students in groups with a relatively large age range: 7–10 years, 11–14 years and 15–17 years. Therefore, we explored the differences in chronological age between patients and students. The mean of the difference scores of age between patients and students after matching was 0.39 years (*SD* = 1.24). Further, the mean age of patients (12.70 years, *SD* =2.71) was significantly higher (*t*(134) = 3.64, *p* < .001, paired *t* test) than in students (*M* = 12.31, *SD* = 2.39). Yet the largest differences (by 2.5–3 years) comprised only five patients (3.7%) who were randomly allocated to students. All other patient–student pairs lay within a range of two years difference, with the majority of 95 cases (70.3%) within a range of one year. We decided not to exclude students who had been in contact with health or school psychological services to avoid an “artificially healthy” comparison group from the general population. The parents of seven matched students (5.2%) responded affirmatively to the question *“Has your child received any help during the last year due to mental health problems or learning difficulties?”.*

### Instruments

#### Quality of life (QoL)

The Kinder Lebensqualität Fragebogen (Questionnaire for Measuring Health-related Quality of Life in Children and Adolescents, revised version, KINDL-R) [25] is a well-established QoL instrument used in several clinical and epidemiological studies. Swedish and Norwegian versions of the Questionnaire were available, and the Danish translation was prepared and approved by the original authors. We used the self-report questionnaire for children and adolescents as well as the proxy version completed by one of the parents. The questionnaire consists of 24 items equally distributed into six subscales: *physical well-being, emotional well-being, self-esteem, family, friends,* and *school*. Each item addresses the child’s experiences over the past week and is rated on a five-point scale (1 = never, 5 = always). Mean item scores are calculated for all subscales and for the *total QoL* scale, which is transformed to a 0–100 scale, with 100 indicating very high QoL. In addition, it provides a disorder-related subscale yielding information about the perception of the disorder burden. We modified the form by adding the sentence “*Concerning your OCD*…” for children, and “*Concerning your child’s OCD*…” for parents to the disorder-related questions to ensure we tracked the informants’ perception of OCD and not a concurrent somatic or other disorder. Psychometric testing of the KINDL-R revealed good scale utilization and scale fit as well as moderate internal consistency [26]. A Norwegian normative study also confirmed satisfactory internal consistency and test–retest reliability [27].

#### Resources and emotional and behavioural problems

The Achenbach Child Behavior Checklist (CBCL) is widely used in Scandinavia for parents to assess competence and emotional and behavioural problems more generally among the children [28], and approved translations are available in all languages. It consists of two sections, one addressing competences and the other assessing emotional and behavioural problems in children aged 6–18 years. The CBCL yields scores for activities, social competence and school performance, which all add to a total summary competence score. The Total Competence scale includes 20 items where parents report the amount and quality of their children’s participation in sports, hobbies, activities, jobs and chores, their friendships, how well the child gets along with others and school functioning. From these items, activities, social and school subscale scores can be calculated. In the present study, only the social and school subscale scores were used. The problem section of the CBCL consists of 113 emotional and behavioural problem items rated on a three-point scale: 0 = “Not true”; 1 = “Somewhat or sometimes true”; 2 = “Very true or often true”. Parents are asked to rate problems the child experienced in the last six months. Two broad dimensions, internalizing and externalizing scales, can be calculated; in the present study, these scales were used as an additional measurement of the psychiatric problem load and correlated with QoL data. Two Norwegian normative studies confirmed satisfactory reliability and validity of the CBCL [29,30].

#### OCD diagnosis and comorbidity

The Kiddie Schedule for Affective Disorders and Schizophrenia-Present and Lifetime version (K-SADS-PL) [31] is a widely used semi-structured interview for diagnostic assessment of DSM-IV [32] psychiatric disorders and subsyndromal symptomatology in children and adolescents. The K-SADS was used to confirm the diagnosis of OCD according to the DSM-IV, and to evaluate comorbidity. The K-SADS-PL was administered by interviewing the parent(s) and the child. Approved translations of the revised version of the K-SADS [33] used in the study were available for all three languages.

#### OCD symptom severity

Symptom severity was assessed with the Children’s Yale-Brown Obsessive Compulsive Scale (CY-BOCS) [34] including Clinical Global Impression (CGI). The CY-BOCS is a semi-structured interview containing checklists of obsessions and compulsions. Scales assessing the severity of obsessions and compulsions separately (range 0–20) are added to a CY-BOCS total score (range 0–40). Finally, a global severity score (CGI) is assigned based on all information gathered during the interview. The checklists and the severity ratings were based on interviews with each child and each parent or adult informant. CGI is a widely used rating scale for clinicians to assess global severity of illness with a score ranging from 0 (“no illness”) to 6 (“serious illness”).

#### Family accommodation

The Family Accommodation Scale (FAS) [35] is a 12-item clinician-rated questionnaire, designed to assess the families’ accommodation to the child’s OCD symptoms. The FAS items measure the extent to which family members provide reassurance or objects needed for compulsions, decrease behavioural expectations of the child, modify family activities or routines, and help the child avoid objects, places or experiences that cause distress. The FAS has demonstrated good psychometric properties including good internal consistency (α = .76, −.80) [35,36], and positive correlations with measures of OCD symptom severity [37] and family discord [35].

#### Duration of symptoms

As a measure of chronicity of symptoms, the duration of OCD was calculated as age minus year of symptom onset. We used this calculation as an indicator of how long the child had struggled with OCD, suggesting that a child suffering from OCD for only a short time might have a different QoL from a child who has struggled with OCD for many years.

#### Socio-economic status

Socio-economic status (SES) was calculated using the highest education level of either mother or father (whichever was highest) assessed as suggested by Hollingshead [38], with scores ranging from 1 to 7 (7 = university education).

### Statistics

All statistical procedures were performed with the Statistical Package for the Social Sciences (SPSS), version 19 (IBM SPSS Inc., Chicago).

In the first five of six subscales of the KINDL-R parent report (n =130), missing values were between 0% and 3.1%; in the *school* subscale, between 7.7% and 9.2%; and in the *disorder* subscale, between 10.8% and 13.1%. In the first five subscales of the KINDL-R children and adolescent’s self-report (n =133), missing values were between 0% and 3.0%; in the *school* subscale, between 5.3% and 6.0%; and in the *disorder* subscale, between 5.3% and 8.3%. Missing values were substituted by the mean according to the KINDL-R manual. In all other instruments, the number of cases with available data for the analyses showed low proportions of cases with missing data, ranging from 0% (CY-BOCS) to 3.7% (FAS). Only the CBCL internalizing scale (11.1%) and externalizing scale (10.4%) had slightly higher proportions of missing data.

QoL total scores and subscales from both patients and caregivers by proxy were compared with those of the matched controls from the general population using paired samples *t* tests. To explore the relation of comorbid disorders in general and QoL, we compared QoL ratings of children with any type of comorbidity with the children with OCD only. To differentiate between disorders such as ADHD, Tourette’s syndrome, autism spectrum disorder, other anxiety disorders and depression, three groups were created based on the presence of comorbidity: (1) OCD only, (2) OCD with ADHD, with tic disorder or with a combination of both, and (3) OCD with another anxiety disorder, with depression or with a combination of both. A general linear model analysis was conducted with the KINDL-R QoL total score and all subscale scores for both children’s self-reports and parent proxy reports as dependant variables. We used Hochberg post hoc tests because of differences in the size of the analysed subgroups. With regard to parent reports of total QoL and the subscale *physical well-being*, we used the Games–Howell test because of significant differences in the variances of the groups. Age and sex proved not to have a significant impact on QoL in the present study (general linear model with QoL as dependent variable), nor did we observe any interaction effects. Age and sex were therefore not included as covariates in the general linear model analysis of the comorbidity groups. Twenty-five cases had mixed or multicomorbidity with three or more diagnoses, making a detailed analysis meaningless, and were subsequently excluded from this set of analyses. With this approach, we excluded six of the seven cases with depression and all with high-functioning autism (HFA). To explore a possible specific relation between the mentioned comorbidities and QoL, we created contrasts between each of them versus the rest of the sample.

To explore possible associations with QoL, other than comorbidity, Pearson’s correlations were conducted for OCD symptom severity (CY-BOCS score/GGI), age of onset, family accommodation and duration of OCD. To explore whether a high symptom load or a threshold of a certain severity was associated with poorer QoL, we compared QoL scores in the group of children with more severe OCD (CY-BOCS score ≥24) with those of the group with less severe OCD (CY-BOCS 16–23). Because of multiple comparisons, the significance level was set to *p* = .01 for all analyses (F-tests and correlations), except for post hoc tests, where *p* was set to .05.

### Ethics

The study was approved by the Regional Committees for Medical and Health Research Ethics in Denmark, Norway and Sweden. All parents gave written informed consent and the permission for their children to participate prior to inclusion in the study.

## Results

### Sample description

Of 155 patients eligible for participation in the study, 22 refused to participate or did not complete the QoL instrument (KINDL-R). Each child and one of the caregivers completed QoL questionnaires resulting in 135 completed forms from either child or/and one of the caregivers (130 parent forms and 133 child forms =128 complete child and parent pairs), yielding a response rate of 86% of eligible children and 84% of the parents (Figure 1). Autism spectrum disorder (ASD) was an exclusion criterion in the main treatment study, but nine individuals with Asperger’s syndrome/HFA were included in a substudy at one site (Trondheim). Complete QoL assessment was available for eight of these nine patients; in the ninth case, only the mother filled in the questionnaire.

The mean age of the participants was 13 years (*SD* 2.7 years, range 7–17). Gender distribution was even with 65 girls (48.1%). Thus, the QoL study sample was representative of the whole sample of the treatment study, where mean age was 12.8 (*SD* 2.7) years, while gender distribution diverged slightly with more girls represented (51.3%). If compared without the nine male participants from the HFA substudy, gender distribution was equal in both samples (51.3% versus 51.6% girls in the QoL study). Ethnicity was primarily Scandinavian; 97% of the participants had one or both parents of Scandinavian origin. The education level of parents was generally quite high (*M* =5.30; *SD* 1.39). The participants in the QoL study were not significantly different from the sample of the main NordLOTS study with regard to SES (*t*(270) = .92; *p* = .357; *M* =5.14, *SD* =1.47).

Comorbidity was common, especially with neuropsychiatric conditions, other anxiety disorders and, to a lesser degree, depression. In fact, only 69 patients (52.3%) had “pure” OCD without any other comorbidity (hereafter named “OCD only”). Eighteen patients (13.6%) met DSM-IV criteria [32] for ADHD and 37 (28%) for tic disorder; 14 had a combination of both. Conduct disorders were diagnosed in six patients (4.5%). Other anxiety disorders were diagnosed in 28 patients (21.2%): Two patients suffered from separation anxiety, 16 from specific phobia (12.1%), eight from social phobia (6.1%), six from generalized anxiety disorder (4.5%) and two from not specified anxiety. Four patients had two, and one patient three, additional anxiety disorders. Depression was found in seven patients (5.3%), with a diagnosis of major depressive disorder in six of them and unspecified depression in one case. No other psychiatric comorbidity was diagnosed. The HFA group (n =9) contributed heavily to the load of comorbidity: Only one of the HFA patients had OCD only, seven had other neuropsychiatric conditions (three tic disorder, two ADHD and two both tics and ADHD) and one patient had another anxiety disorder (specific phobia).

### QoL in OCD compared with the general population

Quality of life as reported by both paediatric patients with OCD and their caregivers was significant lower than QoL reported by the controls (Table 1). Children’s self-report revealed lower scores of total QoL as well as of all subscales except *self-esteem* and *school*. Parent proxy reports had a similar pattern with reduced QoL total score and all subscale scores.

**Table 1:** **Quality of life in children with OCD compared to controls from the general population assessed by self-reports and parent proxy reports**

	**QoL Total score**	**Physical well-being**	**Emotional well-being**	**Self-esteem**	**Family**	**Friends**	**School**
	**Mean (** ***SD*** **)**	**Mean (** ***SD*** **)**	**Mean(** ***SD*** **)**	**Mean (** ***SD*** **)**	**Mean (** ***SD*** **)**	**Mean (** ***SD*** **)**	**Mean (** ***SD*** **)**
**Children with OCD self-reports**	62.40 (13.00)	62.47 (18.07)	63.24 (16.73)	51.24 (20.66)	67.86 (19.49)	65.98 (18.56)	63.55 (19.76)
**General population self-reports**	69.72 (12.38)	70.03 (19.28)	76.53 (14.37)	54.88 (19.34)	76.56 (18.47)	75.98 (18.27)	66.72 (17.73)
p-value	**<.001**	**.003**	**<.001**	.147	**<.001**	**<.001**	.175
**Children with OCD parent reports**	61.63 (13.27)	63.35 (20.19)	63.07 (17.05)	49.16 (16.62)	67.44 (17.04)	62.56 (18.26)	63.98 (19.82)
**General population parent reports**	74.68 (9.97)	78.62 (16.97)	78.58 (12.60)	65.08 (13.56)	73.41 (12.74)	77.49 (13.15)	74.81 (12.73)
p-value	**<.001**	**<.001**	**<.001**	**<.001**	**.009**	**<.001**	**<.001**

n = 121-133 for children’s self-reports.

n = 100-110 for parents’ reports.

**Bold letters** indicate significant p-values.

### Social competence and school functioning

Parents reported a great deal of impairment in the social functioning of children with OCD compared with the general population (Table 2). Also school functioning subscale scores were rated significantly lower in the children with OCD, but not as low as the social competence scores. Because of only approximate normal distribution of the social competence and school functioning scores, we confirmed the results with non-parametric tests. Both scales showed the same highly significant differences (*p* < .001 by Wilcoxon signed-rank test).

**Table 2:** **Social competence and school functioning of children with OCD as rated by parents on the CBCL compared to controls from the general population**

	**Mean**	**Standard deviation**	***t***	***df***	***p***
**Children with OCD:** Social competence score	1.99	1.94	−27.58	103	**< .001**
**General population:** Social competence score	8.79	1.70			
**Children with OCD:** School functioning score	4.20	1.12	−3.90	85	**< .001**
**General population:** School functioning score	4.77	1.07			

Social competence n = 104.

School functioning n = 86.

**Bold letters** indicate significant p-values.

### Comorbidity and QoL

In a first analysis, we compared QoL ratings of children with any type of comorbidity (n = 62) with the children with OCD only (n =68) (Table 3). We found a tendency for lower QoL scores of children’s self-reports in the comorbidity group compared with the children with only OCD. None of the observed differences were statistically significant, though *physical well-being* was nearly significant. For parent reports, we observed a significant impact of comorbidity on QoL with respect to total score and the subscales *physical well-being*, *emotional well-being*, *friends* and *school.*

**Table 3:** **Quality of life in children with OCD only compared to children with OCD and different comorbidities assessed by self-reports and parent proxy reports**

	**QoL Total score**	**Physical well-being**	**Emotional well-being**	**Self-esteem**	**Family**	**Friends**	**School**	**Disorder subscale**
	**Mean (** ***SD*** **)**	**Mean (** ***SD*** **)**	**Mean (** ***SD*** **)**	**Mean (** ***SD*** **)**	**Mean (** ***SD*** **)**	**Mean (** ***SD*** **)**	**Mean (** ***SD*** **)**	**Mean (** ***SD*** **)**
**Children with OCD only self-reports**	64.20 (13.30)	65.38 (16.67)	65.21 (16.07)	53,89 (20.81)	68.29 (20.24)	68.29 (17.12)	63.59 (21.40)	62.14 (17.00)
**Children with comorbidity self-reports**	60.66 (12.14)	59.07 (18.99)	60.96 (17.22)	48.19 (19.38)	67.83 (18.95)	63.51 (19.95)	64.83 (17.71)	66.58 (16.98)
(df)	(1,128)	(1,128)	(1,126)	(1,128)	(1,127)	(1,128)	(1,122)	(1,119)
F -value	2.52	4.07	2.08	2.60	0.02	2.16	0.12	2.07
p-value	.115	.046	.152	.109	.894	.144	.727	.153
**Children with OCD only parent reports**	64.03 (12.75)	66.82 (20.15)	65.07 (16.56)	49.26 (17.26)	68.29 (16.06)	66.36 (17.11)	68.42 (19.96)	60.31
**Children with comorbidity parent reports**	56.26 (12.47)	57.73 (17.34)	57.11 (17.54)	47.33 (16.61)	64.11 (17.35)	54.76 (18.98)	56.84 (19.24)	57.32 (18.55)
(df)	(1,121)	(1,125)	(1,124)	(1,123)	(1,124)	(1,122)	(1,115)	(1,108)
F -value	11.51	11.51	6.85	0.40	1.96	12.78	10.09	0.77
p-value	**.001**	**.008**	**.010**	.527	.164	**.001**	**.002**	.383

n = 64-68 for children with OCD only and n = 53-62 for children with OCD and comorbidity.

**Bold letters** indicate significant p-values.

In a subsequent analysis of two subgroups with specific comorbidity (see above under Methods/Statistics) we again found a tendency towards lower QoL scores of children’s self-reports in both comorbidity groups, but these did not reach statistical significance. Parents reported a significant impact of comorbidity on *friends* subscale scores [*F* (2,97) = 7.65, *p* = .001]. Subsequent post hoc analysis also showed significantly higher QoL on the subscale *friends* for the OCD only group (*M* = 66.36; *SD* = 17.11, n = 68) compared with the group with OCD and comorbid neuropsychiatric disorders (*M* = 47.79; *SD* = 20.37, n = 17, *p* = .001). Parents reported a borderline significant impact on total QoL scores (*F* [2,97] = 4.51, *p* = .013). Post hoc testing showed higher QoL total scores for the OCD only group (*M* = 64.03; *SD* = 12.75, n = 68) than for the group with OCD and comorbid neuropsychiatric disorders (*M* = 55.04; *SD* = 10.31, n = 17, *p* = .013). Further, we found non-significant tendencies for the OCD only group to show higher QoL than the group with neuropsychiatric disorders on three additional subscales, as reported by the parents: *physical well-being* (*F* [2,100] = 3.29, *p* = .041), *emotional well-being* (*F* [2, 100] =2.98, *p* = .055) and *school* (*F* [2,92] = 3.01, *p* = .054).

Children with tic disorder did not differ significantly in QoL from any other group, irrespective of other comorbidities. Parent ratings showed a tendency to score children with tics lower on total QoL and on different subscales (*emotional well-being, friends* and *school)*, but this difference did not reach statistical significance. Patients with comorbid depressive disorder (n = 7) and their parents reported lower total QoL and scores on the subscales *physical well-being*, *emotional well-being* and *school* than all other patients, but again, without reaching significance levels. Children with HFA and OCD and their parents reported similar QoL scores to the other patients on all scales.

### Symptom severity

For children’s self-reports, we found a significant (*p* < .001) negative association between symptom severity expressed by CY-BOCS scores and the KINDL-R *disorder* subscale only (Table 4). For parent proxy reports, a significant association was found between CY-BOCS score and QoL total score (*r* = −.28) and four subscales (*emotional well-being, friends, school* and the *disorder* subscale score) (*p* < .01). Although significant, the correlations were low to moderate (range –.24 to –.45). Children with more severe OCD (CY-BOCS score ≥24, n = 81) and their parents reported significantly lower scores on the *disorder* subscale (children: *M* = 59.22, *SD* = 16.30 and parents: *M* = 54.16, *SD* = 16.38) than children with less severe OCD (n = 51) (*M* = 71.90, *SD* = 15.63; parents *M* = 67.05, *SD* = 17.43, *p* < .001, not shown in table). In addition, children’s ratings showed non-significant tendencies in the same direction for total QoL (*p* = .027), *emotional well-being* (*p* = .030) and *self-esteem* (*p* = .032). Parents’ reports confirmed this pattern with non-significant tendencies to lower scores for total QoL (*p* = .032) and the subscale *emotional well-being* (*p* = .016).

**Table 4:** **Pearson correlations between different parameters and quality of life assessed by children’s self-reports and parent proxy reports**

**n =113-135**	**QoL Total score**	**Physical well-being**	**Emotional well-being**	**Self-esteem**	**Family**	**Friends**	**School**	**Disorder subscale**
**OCD severity: CYBOCS score**								
QoL Self-report	- .19	- .16	- .17	- .16	- .15	- .12	- .06	- .38
p-value	.025	.067	.048	.073	.096	.188	.54	**<.001**
Parents report	- .28	- .17	- .24	- .13	- .19	- .26	- .24	- .45
p-value	**.001**	.053	**.006**	.148	.033	**.003**	**.010**	**<.001**
**Family Accommodation: FAS score**								
QoL Self-report	- .10	- .16	- .15	- .07	- .15	.00	.10	- .14
p-value	.253	.075	.092	.414	.087	.974	.273	.143
Parents report	- .40	- .29	- .39	- .26	- .30	- .30	- .21	- .49
p-value	**<.001**	**.001**	**<.001**	**.004**	**.001**	**.001**	.026	**<.001**
**Age of onset of OCD: Year**								
QOL Self-report	- .02	.15	- .02	- .08	- .1	- .03	- .11	- .15
p-value	.786	.077	.821	.355	.909	.708	.230	.106
Parents report	.08	.16	.10	.10	.04	- .03	- .01	- .004
p-value	.353	.077	.258	.257	.635	.750	.950	.968
**Duration of OCD: In years**								
QoL Self-report	- .18	- .13	- .16	- .09	- .08	- .09	- .20	- .12
p-value	.036	.147	.064	.323	.369	.307	.024	.180
Parents report	- .19	- .29	- .14	- .13	- .06	- .05	- .22	- .07
p-value	.032	**.001**	.126	.140	.522	.564	.019	.460
**CBCL internalizing score**								
QoL Self-report	- .35	- .34	- .39	- .22	- .14	- .25	- .09	- .22
p-value	**<.001**	**<.001**	**<.001**	.015	.130	**.007**	.358	.022
Parents report	- .59	- .51	- .57	-.32	- .32	- .44	- .39	- .47
p-value	**<.001**	**<.001**	**<.001**	**.001**	**.001**	**<.001**	**<.001**	**<.001**
**CBCL externalizing score**								
QoL Self-report	- .33	- .21	- .31	- .22	- .31	- .21	- .05	.01
p-value	**<.001**	.020	**.001**	.014	**.001**	.023	.636	.910
Parents report	- .50	- .24	- .48	- .17	- .56	- .40	- .30	- .33
p-value	**<.001**	**.008**	**<.001**	.067	**<.001**	**<.001**	**.002**	**.001**

**Bold letters:** Correlation is significant at the .01 level (2-tailed).

### Family accommodation

In children’s self-reports, we found no association between levels of family accommodation expressed by the FAS score and QoL (Table 4). In parent proxy reports, significant low to moderate negative correlations were found between FAS score and QoL total score (*r* = −.40) and all subscales (*p* < .01), except the *school* subscale, which showed a non-significant (*p* = .026) tendency only.

### Age of onset and duration of OCD

There was no association between QoL and age of onset of OCD. Parents’ reports showed the duration of OCD symptoms was significant with regard to *physical well-being* yielding a moderate negative correlation (*r* = −.29, *p* = .001). Total QoL and *school* reported by parents and children showed a non-significant tendency (Table 4) with low negative correlations.

### CBCL internalizing and externalizing problems

The CBCL internalizing score showed overall significant correlations with QoL in children’s and parent proxy reports (*p* < .01): Parents’ reports of QoL total score and all subscales were negatively correlated with the CBCL internalizing score (low to medium correlations). In children’s reports, the correlation was significant for QoL total score and three subscales only (*physical well-being, emotional well-being* and *friends*). For the externalizing problems, a pattern similar to that shown for internalizing problems emerged. We found significant low to moderate negative correlations between QoL and CBCL externalizing score for parents’ reports of total QoL and all subscales except *self-esteem.* For children’s reports, the total QoL, *emotional well-being* and *family* subscales were significantly (*p* < .01) negatively correlated with externalizing scores, with the correlations being somewhat lower than for the parents’ report.

## Discussion

We found a clear reduction of quality of life in children with OCD, which affected total QoL measurements and most of the subdomains reported by both patients and their caregivers. Lack and colleagues [21] underlined the relevance of examining QoL in children with OCD as a source of useful information for treatment planning. Our findings are in accordance with this study [21], corroborating their results of heavily impaired QoL in children with OCD with a larger sample size and a methodologically stringent design.

We compared parents’ ratings of the children with OCD on the CBCL social competence and school functioning scales with the general population to add the parents’ more “objective” rating of functional aspects as an external perspective to the QoL analysis, which measures by definition subjective aspects of well-being. Parents reported markedly impaired social functioning of the children with OCD compared with the general population. School performance was also reduced but to a lesser degree. This result seems to be in accordance with the clinical experience that OCD symptoms might have a more profound impact on social life than on school performance.

Concerning comorbidity, we found considerably reduced QoL in the comorbidity group compared with children with “pure” OCD, at least in the view of their caregivers. In children’s reports, we observed the same tendency but without reaching significance levels. That K-SADS-diagnosed DSM-IV comorbidity showed significantly reduced QoL only in parent reports might be due to the general tendency of children to rate their QoL with a more optimistic view than their parents, as seen in other studies. In studies of parent–child agreement in QoL, parents of children in non-clinical samples tend to report higher QoL scores than their children [39], while parents of children with disorders tend to under-estimate QoL compared with the children’s ratings. In Lack and colleagues’ study [21], parents generally rated QoL as lower than the youths did. We agree with the authors’ suggestion that young people may minimize the impact on QoL of their condition. Another possible explanation could be that the numbers of children with a categorical DSM-IV comorbidity diagnosis might be too low to reach significance levels in children’s QoL reports. The disadvantage of categorical DSM-IV diagnoses is that patients on a subclinical level, who fall short of fulfilling diagnostic criteria, are not taken into account despite having considerable problems. Accordingly, on the symptom level, CBCL internalizing and externalizing problems showed a clear moderate negative association with QoL. The dimensional assessment approach of the CBCL, summarizing symptoms into quantitative externalizing and internalizing problem scores, might have been more sensitive than the diagnostic categorical approach to detect QoL impairment associated with comorbidity in all domains.

Almost half of the OCD patients in our sample were also affected with a variety of other disorders, and it was difficult to disentangle the relationships between different comorbidities and QoL, especially as statistical power was reduced by the low numbers in the comorbidity subgroups. In our analysis of two subgroups with specific comorbidity (ADHD, tic disorder or both, and anxiety disorder, depression or both) compared with the children with only OCD, we again found the tendency for lower QoL to be reported by the children in both comorbidity subgroups. On the other hand, parents reported significantly worse total QoL and relation to *friends* for the group with OCD and neuropsychiatric disorders than for the OCD only group, while comorbid anxiety disorders were not associated with worse QoL.

Both ADHD and Tourette’s syndrome are associated with poor QoL in children and adolescents [40-42]. The presence of both OCD and ADHD is associated with reduced QoL in young people with Tourette’s syndrome [43,44]. While confirming the additional negative impact of a comorbid neuropsychiatric disorder on QoL in paediatric patients with OCD, we were not able to differentiate the potential effects of ADHD versus Tourette’s syndrome because of the sample size and overlapping comorbidity. Severe depression with suicidal ideation was an exclusion criterion in the NordLOTS study. This could be one of the reasons why we had a comparatively low prevalence of depression in our sample. The seven patients with depressive disorder and their caregivers reported a non-significant tendency towards lower QoL compared with the other patients. One must bear in mind that six of the seven patients with depression had one or more other comorbidities; the reduced QoL may well be a reflection of the general high load of comorbidity. On the other hand, the additive impact of comorbid depression on QoL is well documented in previous studies, at least in adults [17,18].

Children with high-functioning autism and their caregivers rated QoL in the same range as the other patients. Previous papers have discussed whether subjectively perceived QoL can be reliably measured in children with ASD because of differences in perception [45]. Shipman and colleagues [46] provided preliminary evidence that adolescents with ASD were able to report on their QoL in a valid and reliable manner. However, children with ASD and comorbid OCD are reported to have poorer insight into the exaggerated nature of their obsessions and they may perceive their compulsions as less egodystonic [47,48]. OCD symptoms usually have a great impact on social life, but children with ASD may be prone to report better QoL because of their reduced interest in social activities. Both factors may contribute to less impaired QoL perception. On the other hand, a possible explanation could be that OCD affects QoL to such an extent that there is no room for further QoL reduction in the HFA patients. However, the number of children with ASD included in our study does not allow us to draw firm conclusions.

Finally, we explored the significance of other factors for the perceived QoL, such as severity of OCD, the family’s adjustment to the symptoms, age of onset and duration. As expected, we found a negative association between symptom severity and QoL, mainly reported by parents. Although significant, the correlations were low to moderate. In children’s self-reports, the negative association between symptom severity and QoL emerged in the *disorder* subscale only. To test the hypothesis that a threshold of a certain severity must be crossed to have a stronger impact on QoL, we compared QoL ratings of children with more severe OCD with the group with less severe OCD. The majority of children in our sample had more severe OCD with CY-BOCS scores of 24 or above. These children and their parents reported significantly lower scores on the *disorder* subscale than children with less severe OCD. The *disorder* section of the KINDL-R reflects the children’s subjective perception of how well they are able to cope with their OCD problems. That more severe symptoms were associated with a perception of reduced coping abilities is consistent with our expectation. We also found low to moderate inverse associations between levels of family accommodation and QoL total score and all subscales, except for the *school* subscale in parents’ reports. As the Family Accommodation Scale score by definition reflects the involvement of the parents in their children’s OCD symptoms, this result was expected. The finding that parents’ perceptions of QoL affect total score and all other domains except *school* is consistent with the clinical observation that OCD symptoms are often more dominant in the home environment than at school. Alternatively, it may simply reflect the fact that parents are less involved in OCD while their children are at school. There was no association between QoL and age of onset of OCD. Duration of OCD was significant in the parents’ report only with regard to *physical well-being.* Surprisingly, the duration of symptoms did not emerge as an important factor. A possible explanation could be that both children and parents adjust to the disorder burden over time, and this is reflected in their perception of QoL.

Both children’s and parents’ reports of QoL total score and most of the subdomains were inversely associated with the CBCL internalizing and externalizing problems. The correlation between severity of symptoms, family accommodation and load of comorbid internalizing and externalizing problems, and reduced QoL underlines the importance of having these factors in mind as part of the assessment procedure of children with OCD. Although all of these factors were correlated with QoL, none of them showed a high correlation, indicating the separate value of assessing QoL. This finding seems to support our view that QoL evaluation covers different and important aspects of the disorder, which are not accounted for in the frame of symptomatic and functional assessment only.

### Limitations

Because of the homogeneous socio-demographic factors and similarities in culture and language of the population in the Nordic countries, our sample consisted mostly of relatively well-educated families of Caucasian origin. For example, the inability to understand one of the Nordic languages was an exclusion criterion. This represents a clear limitation to the generalization value for other populations. Although our study was based on a relatively large sample of paediatric patients with OCD, some subgroups of comorbid disorders were small, leading to problems of statistical power, which limited an analysis of each single comorbid disorder group. To overcome this limitation in further research, much larger samples are needed, which would demand not only regional multicentre studies but also collaboration across different countries. Another limitation lies in the cross-sectional design of the study; this design generally does not allow conclusions to be drawn about causality and effect of the observed associations between QoL and the examined factors.

## Conclusions

QoL and social competence are significantly reduced in children and adolescents with OCD compared with the general population. OCD with comorbidity showed the lowest QoL compared with OCD without any comorbidity. Severity and duration of symptoms, family accommodation and comorbid internalizing and externalizing problems were associated with reduced QoL, but with low to moderate correlations only. The assessment of QoL beyond symptoms and function in children with OCD has been shown to be reliable and informative. Thus, we recommended QoL as an additional outcome measure in the treatment plans of children with OCD.
